# Tunable asymmetric spin wave excitation and propagation in a magnetic system with two rectangular blocks

**DOI:** 10.1038/s41598-021-02967-9

**Published:** 2021-12-21

**Authors:** Dongpyo Seo, S. Hwang, Byungro Kim, Yeonhee Yang, Seungha Yoon, B. K. Cho

**Affiliations:** 1 School of Materials Science and Engineering, Gwangju Institute of Science and Technology (GIST), Gwangju, 61005 Republic of Korea; 2 Green Energy & Nano Technology R&D Group, Korea Institute of Industrial Technology (KITECH), Gwangju, 61012 Republic of Korea

**Keywords:** Magnetic properties and materials, Spintronics

## Abstract

Asymmetric spin wave excitation and propagation are key properties to develop spin-based electronics, such as magnetic memory, spin information and logic devices. To date, such nonreciprocal effects cannot be manipulated in a system because of the geometrical magnetic configuration, while large values of asymmetry ratio are achieved. In this study, we suggest a new magnetic system with two blocks, in which the asymmetric intensity ratio can be changed between 0.276 and 1.43 by adjusting the excitation frequency between 7.8 GHz and 9.4 GHz. Because the two blocks have different widths, they have their own spin wave excitation frequency ranges. Indeed, the spin wave intensities in the two blocks, detected by the Brillouin light scattering spectrum, were observed to be frequency-dependent, yielding tuneable asymmetry ratio. Thus, this study provides a new path to enhance the application of spin waves in spin-based electronics.

## Introduction

There has been great interest in spin-based electronics because of its high potential for application to new electronic devices, such as signal processing and data storage. One of the data storage devices is magnetic random access memory (MRAM), which manipulates spin states using spin transfer torque^1–3^ or spin orbit torque^4–6^. In signal transfer and processing applications, magnonics, which use spin waves as information carriers, have attracted considerable attention. Due to the absence of charge carrier transportation, spin wave devices have some advantages such as low power consumption and high processing speed^7^. In addition, many studies have proposed magnonic switches, transistors, and logic devices in recent years^8–12^.

One of the interesting properties of spin waves in device applications is the nonreciprocal propagation of spin waves. The magnetostatic surface spin wave (MSSW), which moves coplanar with and perpendicular to the magnetized direction, propagates with directional dependent intensity due to the asymmetry of the induced field. Such characteristics are called spin wave nonreciprocity. Thus, spin wave nonreciprocity can provide additional degrees of freedom for the control of signal propagation^13–17^. Spin wave nonreciprocity has large potential for applications in switches and logic devices due to selectively unidirectional spin wave propagation^12,18^, while in devices that require bidirectional signal propagation, the spin wave intensity should be the same in both directions^19^.

Spin wave nonreciprocity is quantified by comparing the spin wave intensities in different directions. Manipulation of the intensity ratio, defined as the nonreciprocity ratio, is the main target in recent studies^20,21^. Various parameters have been utilized to control the spin wave nonreciprocity: the frequency of microwaves, magnitude of the external field, excitation antenna width and film thickness^13,16^. The nonreciprocity ratio values were reported to be small (0.4–0.7). Moreover, the wave propagation direction with a larger amplitude could not be controlled. To overcome these problems, Kwon et al. studied a bilayer of tantalum and permalloy and found that it gives a large value of nonreciprocity (~ 60) and a preferred direction of spin propagation^14^. Deorani et al. also suggested a system with two antennas on both sides of a permalloy film, and obtained a nonreciprocity ratio larger than 50^19^. However, magnetic film systems have no flexibility to change the nonreciprocity ratio and the wave propagation direction once the system is fabricated. It would be of great interest to find a way to control the intensity ratios and direction in a system.

Here, we suggest two bar-type permalloy(Ni_80_Fe_20_) blocks that have different widths and are located on a line with a narrow separation (~ 0.3 μm). Because the two blocks have their own frequency range for spin excitation, the excitation characteristics would have a significant dependence on the RF frequency. Thus, it is found that both the spin wave asymmetry ratio and propagation direction can be controlled by the applied excitation RF current.

## Experimental details

**Figure 1:**
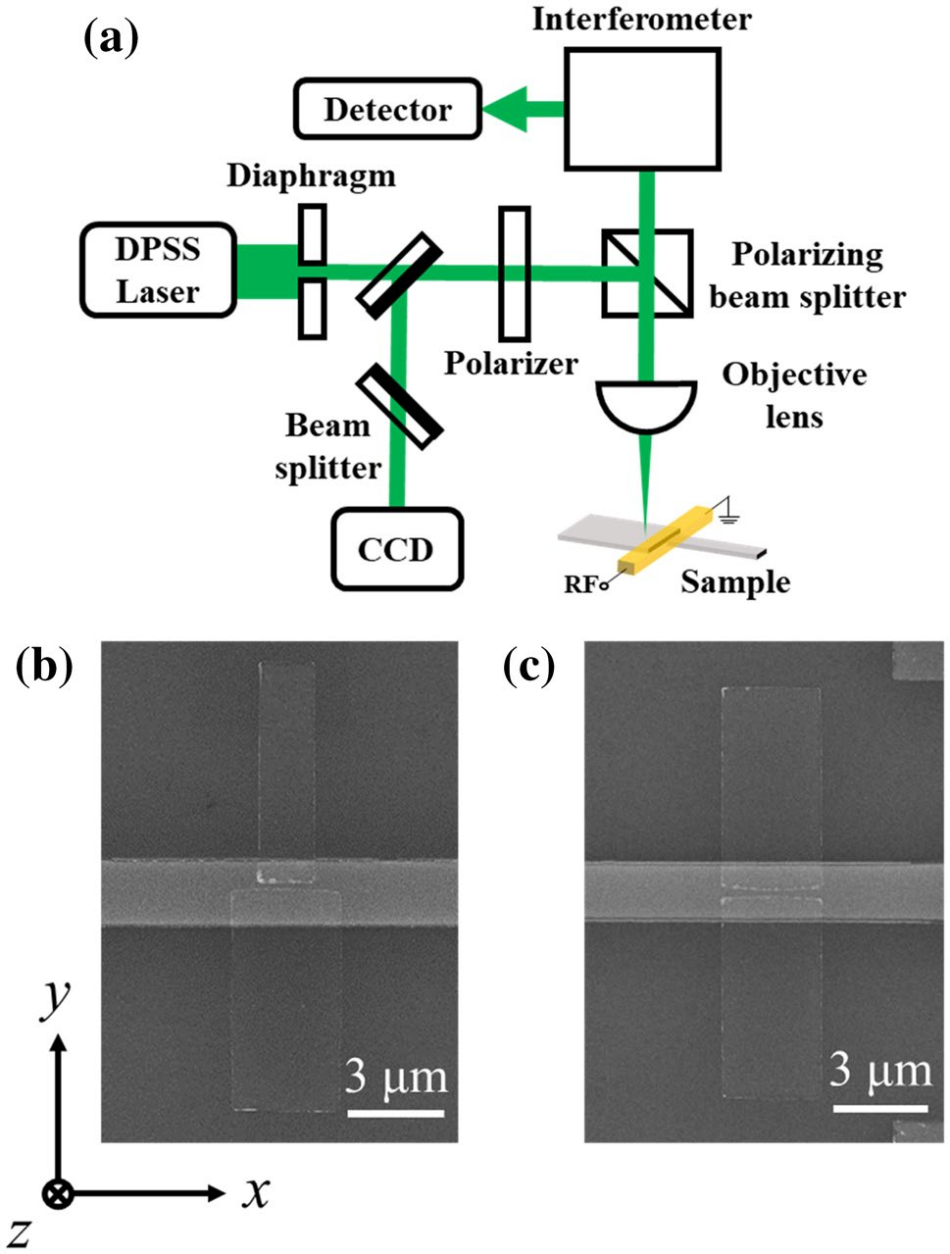
(**a**) Schematic diagram of the BLS measurement system. Scanning electron microscopy image of the magnetic blocks, separated by small gap and excitation antenna: (**b**) The two blocks have different widths. (**c**) The two blocks have identical width.

Spin wave propagation was detected using a Brillouin light scattering (BLS) spectroscopy system. The BLS spectroscopy is an optical tool that uses inelastic scattering of photons with some quasiparticles, similar to Raman spectroscopy, working between photons and phonons. The BLS spectroscopy has the advantage of investigating excitations in the GHz frequency regions where, in general, spin waves prevail^22^. A schematic diagram of the BLS measurement setup is shown in Fig. 1a. To obtain a high-resolution image of spin wave propagation, a spatially resolved micro-BLS (μ-BLS) system is used. An Ar^+^ ion laser (*λ* = 514.5 nm) is used as the monochromatic light source. The scattered light from the sample was transferred to a Fabry–Perot interferometer with two etalons. High contrast of the signal is obtained as the light is transferred through both etalons. The spatial interval of the measurement is set to 100 and 200 nm for the *x-* and *y-*directions, respectively. The sample surface and laser spot were visualized by a CCD camera for real-time measurement observation.

SEM images of samples A and B are shown in Fig. 1b,c, respectively. Sample A consists of two rectangular blocks one with a width of 1.61 μm and the other with a width of 3.14 μm, which have a gap of 0.3 μm between them. Sample B consists of two identical rectangular blocks with a width of 3.09 μm and the same gap. The blocks are multilayers of Ta(3 nm)/Ni_80_Fe_20_(20 nm)/SiO_2_(5 nm). The RF microwave current antenna covers the gap across the two blocks, as shown in Fig. 1. The antenna is a stack of SiO_2_(40 nm)/Ta(3 nm)/Cu(150 nm)/Ta(5 nm) layers. Blocks and antennas are patterned by an e-beam lithography process.

A spin wave is generated with an applied field of ${H}_{x}=800$ Oe along the *x*-direction. An RF microwave current with a power of $P=100$ mW flows through the antenna in the same direction as the external field.

## Results and discussion

The dispersion relation is calculated by considering both dipole–dipole and exchange interactions^23,24^. The spin wave frequency of mode number *n* on a thin film can be expressed as^24^

1 $${\omega }_{n}^{2}=\left({\omega }_{H}^{n}+\alpha {\omega }_{M}{k}^{2}\right)\left[{\omega }_{H}^{n}+\alpha {\omega }_{M}{k}^{2}+{\omega }_{M}F\left(kL\right)\right],$$

where $\alpha$ is the exchange constant, ${k}^{2}={k}_{nx}^{2}+{k}_{y}^{2}$ is an in-plane component of the wave vector, ${\omega }_{H}^{n}={\omega }_{H}+{\omega }_{M}{N}_{x}\left(x\right)$, ${\omega }_{H}=\gamma {H}_{ex}$, ${\omega }_{M}=4\pi \gamma {M}_{s}$, $\gamma$ is the gyromagnetic ratio, ${M}_{s}$ is the saturation magnetization, and ${H}_{ex}$ is an external field. The demagnetization factor ${N}_{x}$ is

2 $${N}_{x}=\frac{1}{\pi }\left[\mathrm{arctan}\frac{t}{2x+w}-\mathrm{arctan}\frac{t}{2x-w}\right],$$

where $t$ is the thickness and $w$ is the width of the film. In Eq. (1), $F(kL)$, which represents a quantized matrix element of the dipole–dipole interaction can be written as

3 $$F\left(kL\right)=1+P\left(kL\right)\left[1-P\left(kL\right)\right]\left(\frac{{\omega }_{M}}{{\omega }_{H}^{n}+\alpha {\omega }_{M}{k}^{2}}\right)\times \left(\frac{{k}_{y}^{2}}{{k}^{2}}\right)-P\left(kL\right)\left(\frac{{k}_{nx}^{2}}{{k}^{2}}\right),$$

where $$P\left(kL\right)$$ is

4 $$P\left(kL\right)=1-\frac{1-\mathrm{exp}\left(kL\right)}{kL}.$$

The exchange constant and gyromagnetic ratio of the permalloy used in this calculation are $\alpha =2.7975\times {10}^{-17}$ m^2^ and $\frac{\gamma }{2\pi }=2.8\times {10}^{-3}$ GHz/Oe, respectively. The measured saturation magnetization is 816 Oe.

**Figure 2:**
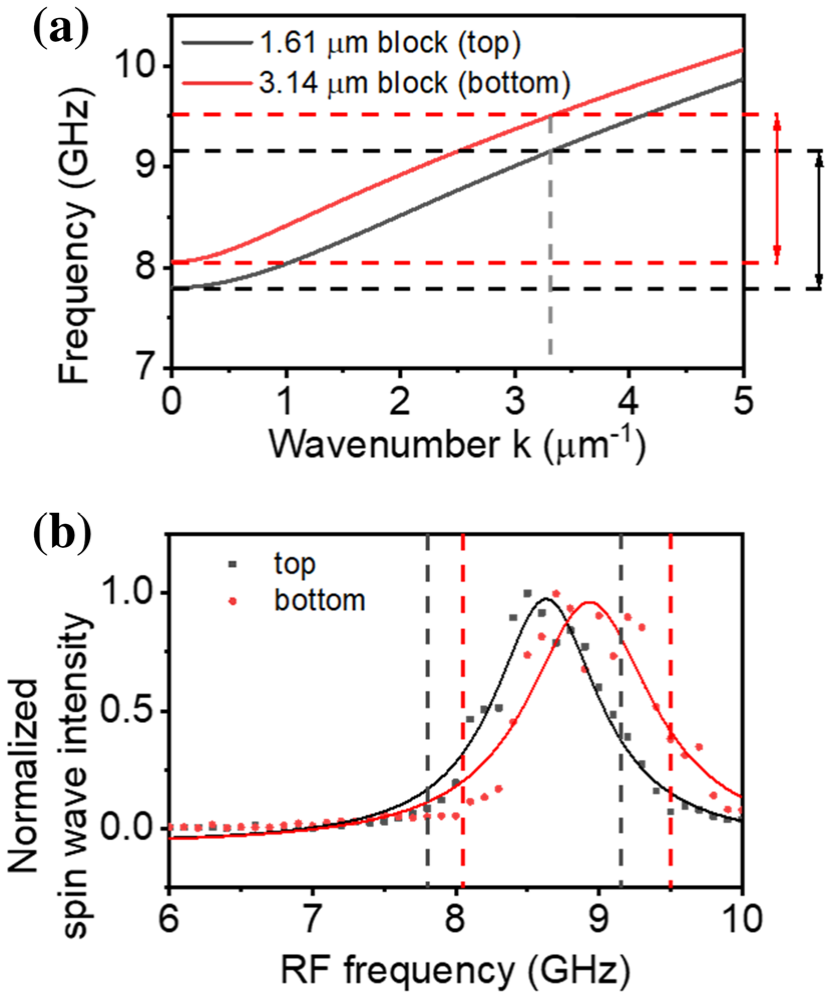
(**a**) Dispersion relation of the spin wave with mode number *n* = 1 for NiFe blocks 1.61 μm wide (black line) and 3.14 μm wide (red line). (**b**) Normalized spin wave intensity of the two NiFe blocks in terms of the excitation RF frequency.

The calculated dispersion relations of the spin wave with mode number *n* = 1 for the blocks are shown in Fig. 2a. It shows that the dispersion has a clear dependence on the block width, i.e., the upper dispersion curve (in red) for the block with a width of 3.14 μm and the lower curve (in black) for that with a width of 1.61 μm. Thus, the ferromagnetic resonance frequency in the wider block is found to be 8.06 GHz, and that for the narrower block is 7.80 GHz. It is known that the spin wave wavelength should be larger than the width of the microwave antenna for efficient excitation^15^. Considering the 1.9 μm width antenna used in this study, we found that the spin wave amplitude with a wavevector larger than 3.31 μm^−1^ diminishes significantly. As a result, the spin wave frequency, which is allowed in the narrower block, is found in a range of 7.80 ≤ *ω* ≤ 9.15 GHz (denoted by a black arrow in Fig. 2a). The allowed frequency for the wider block is also found in a range of 8.06 ≤ *ω* ≤ 9.51 GHz (denoted by a red arrow).

The measured BLS intensities for the two blocks are plotted in terms of the frequency in Fig. 2b. It is clear that the BLS intensity is strong in the frequency ranges for each block and decreases rapidly outside the ranges. Thus, significant asymmetric intensity is expected near both ends of the frequency ranges. If the RF frequency is larger than 9.15 GHz, the spin wave in the wider block is excited while the spin wave in the narrower block is negligible. The opposite situation occurs if the RF frequency is smaller than 8.06 GHz. This means that the spin waves in the field ranges of 7.80 GHz ≤ *ω* ≤ 8.06 GHz and 9.15 GHz ≤ *ω* ≤ 9.51 GHz are excited only in the narrower and wider blocks, respectively. The waves propagate in a specific direction, i.e., upward in the narrower block and downward in the wider block. On the other hand, in a frequency range of 8.06 ≤ *ω* ≤ 9.15 GHz, the spin waves are excited in both blocks at the same time and propagate along both directions. This implies that frequency modulation can be a valid way to manipulate the spin wave excitation and propagation in this geometry.

**Figure 3:**
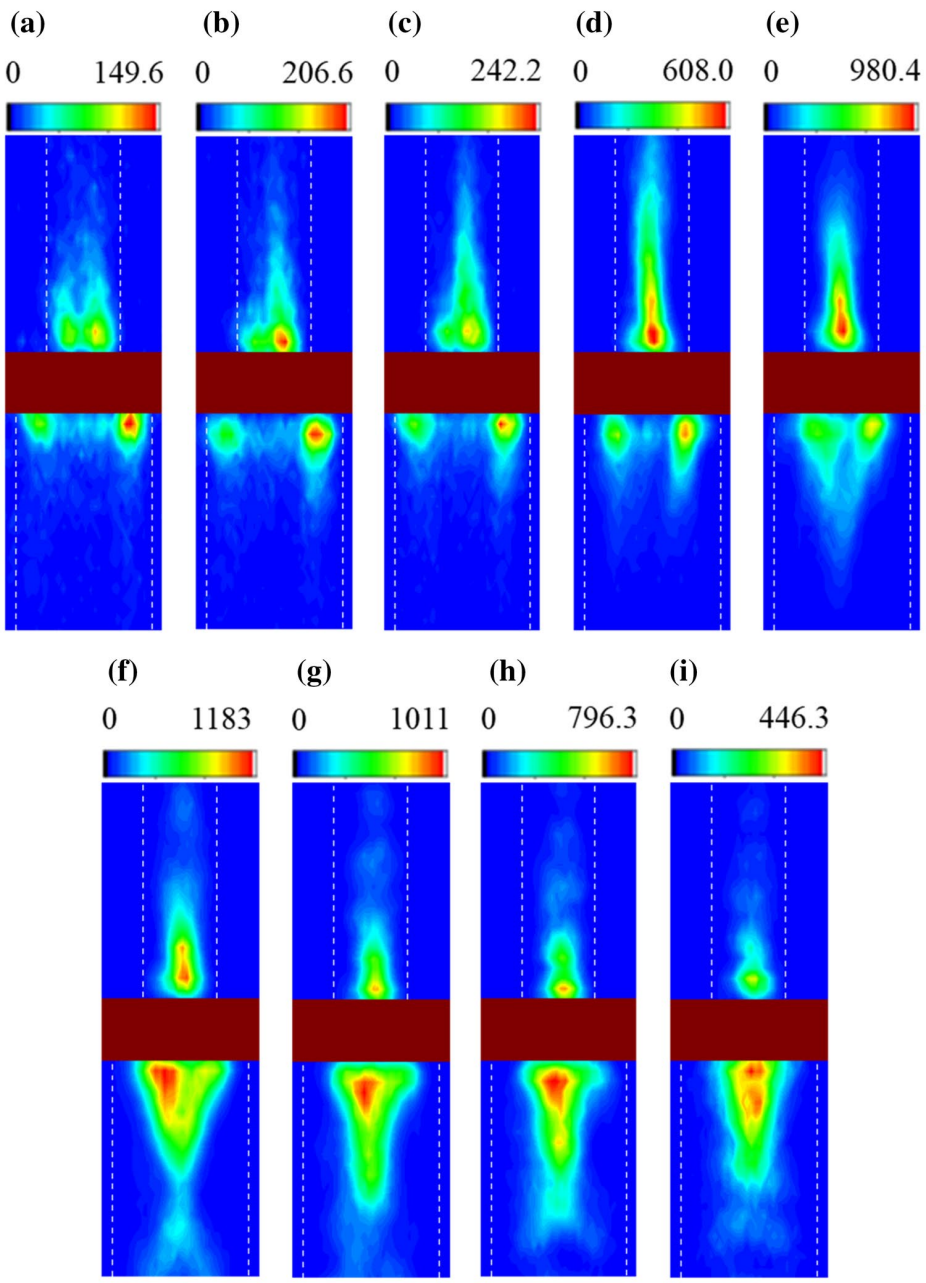
Spatial profiles of Brillouin light spectrum with various RF frequencies in the two blocks: (**a**) 7.6 GHz (**b**) 7.8 GHz (**c**) 7.9 GHz (**d**) 8.2 GHz (**e**) 8.4 GHz (**f**) 8.8 GHz (**g**) 9.0 GHz (**h**) 9.2 GHz and (**i**) 9.4 GHz.

To observe spin wave propagation, spatial profiles of the spin waves in sample A with various frequencies are observed and plotted in Fig. 3. Spin wave intensities collected by BLS measurement system at each point are mapped. At a frequency of 7.6 GHz, no spin wave excitation is observed except for the spin wave of the edge mode in the wider block (Fig. 3a). At the frequencies of 7.8 GHz and 7.9 GHz, the spin waves are observed to be excited only in the narrower block (Fig. 3b,c). As the frequency increases (Fig. 3d,e), the spin waves in the wider block, as well as in the narrower block, start to be excited. At a frequency of 8.8 GHz (Fig. 3f), the BLS spectrum clearly shows the spin wave excitation in both blocks with almost the same intensity. With a further increase in frequency (Fig. 3g–i), the BLS intensity in the narrower block decreases while the intensity in the wider block remains strong. The observations in Fig. 3 are consistent with the discussion on spin wave excitation based on the dispersion relation. Asymmetric spin wave excitations are clearly observed depending on the frequency. This means that in the two block system (sample A), the asymmetric spin wave excitation and propagation are manipulated by frequency variation.

**Figure 4:**
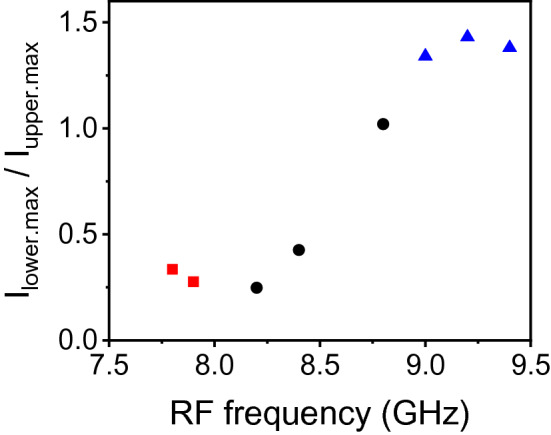
Intensity ratio (I_lower.max_/I_upper.max_) of the Brillouin light scattering spectrum in Fig. 3, where I_lower.max_ and I_upper.max_ are the maximum intensities in the wider and narrower blocks, respectively. Red squares represent spin wave excitation only in the narrower block, blue triangles excitation only in the wider block and black circles excitation in both blocks.

From the data in Fig. 3, the spin wave intensity ratio between the narrower and wider blocks, defined as the asymmetry ratio, is plotted in Fig. 4. The data, indicated by red square symbols, represent the spin wave excitation in the narrower block and wave propagation upward. The data, represented by black circle symbols, are for the excitation in both blocks and bidirectional propagation, and those by blue triangles are for the excitation in the wider block and propagation downward. The asymmetry ratio changes from 0.276 to 1.43 in a system (sample A in this study), and the wave propagation direction also changes with the variation of frequency.

**Figure 5:**
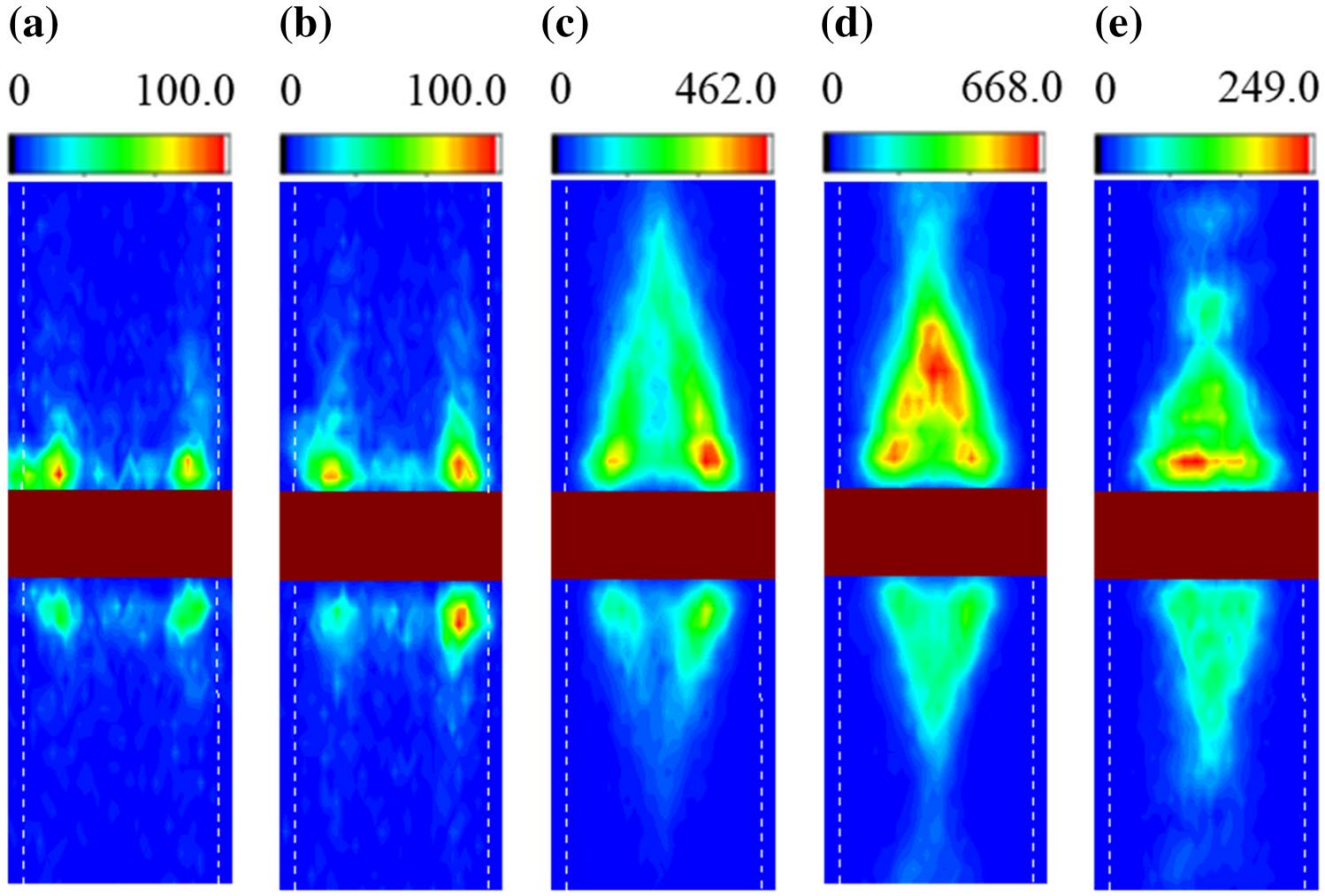
Spatial profiles of Brillouin light scattering spectrum with various RF frequencies in the two blocks with identical dimensions: (**a**) 7.6 GHz (**b**) 7.9 GHz (**c**) 8.4 GHz (**d**) 8.8 GHz and (**e**) 9.4 GHz.

For comparison, spatial profiles of spin wave excitation in the two blocks with the same dimension (sample B) are plotted in Fig. 5. At low frequencies of 7.6 GHz and 7.9 GHz, no excitation of the main mode wave, other than the edge mode, is observed in either the upper or lower blocks (Fig. 5a,b). At the frequencies of 8.4 GHz, 8.8 GHz and 9.4 GHz (Fig. 5c–e), the spin excitation in both blocks is observed with slight intensity differences. The intensity difference is likely due to asymmetric field application, induced by the antenna, which is also observed in other studies^15,16,19^. Thus, the characteristics in sample B cannot be utilized for the control of asymmetric spin wave excitation and propagation, as reported in previous research^16^.

## Conclusion

We investigated the spin wave characteristics in a magnetic system of two blocks, which have different width dimensions (1.61 and 3.14 μm) and are separated with a small gap (0.3 μm). We found that the spin wave excitation and its propagation can be controlled by adjusting the excitation frequency. The spatial BLS spectrum shows that the spin wave is excited only in the narrower and wider blocks at low ($\approx$ 7.9 GHz) and high frequencies ($\approx$ 9.4 GHz), respectively, and in both blocks at frequencies ($\approx$ 8.8 GHz) between them. As a consequence, the spin wave propagation direction is determined to be unidirectional downward and upward at low and high frequencies, respectively, and bidirectional at the frequencies between them. These observations are found to be consistent with the analytical dispersion relations for the two blocks. The asymmetry ratio, defined as the BLS intensity ratio of the spin wave in the blocks, is determined to be in the range between 0.26 and 1.43, depending on the excitation frequency.

The two-block system shows a new function of tunable nonreciprocal effects of asymmetric spin wave excitation and propagation without structural modification. Compared to the pre-existing system, the discovery in this study affords an additional degree of freedom in signal processing. Our system will provide a new possibility for the applications of spin-based electronics, such as spin logic devices, switch devices, and spin wave-based data transporting systems.

## References

[1] Katine JA, Albert FJ, Buhrman RA, Myers EB, Ralph DC (2000). Current-driven magnetization reversal and spin-wave excitations in Co/Cu/Co pillars. Phys. Rev. Lett..

[2] Huai Y, Albert F, Nguyen P, Pakala M, Valet T (2004). Observation of spin-transfer switching in deep submicron-sized and low-resistance magnetic tunnel junctions. Appl. Phys. Lett..

[3] Wang KL, Alzate JG, Amiri PK (2013). Low-power non-volatile spintronic memory: STT-RAM and beyond. J. Phys. D Appl. Phys..

[4] Zhang S (2000). Spin Hall effect in the presence of spin diffusion. Phys. Rev. Lett..

[5] Miron IM, Garello K, Gaudin G, Zermatten P, Costache MV, Auffret S, Bandierra S, Rodmacq B, Schuhl A, Gambardella P (2011). Perpendicular switching of a single ferromagnetic layer induced by in-plane current injection. Nature.

[6] Liu L, Lee OJ, Gudmundsen TJ, Ralph DC, Buhrman RA (2012). Current-induced switching of perpendicularly magnetized magnetic layers using spin torque from the spin Hall effect. Phys. Rev. Lett..

[7] Kruglyak VV, Demokritov SO, Grundler D (2010). Magnonics. J. Phys. D Appl. Phys..

[8] Chumak AV, Serga AA, Hillebrands B (2014). Magnon transistor for all-magnon data processing. Nat. Commun..

[9] Cheng R, Daniels MW, Zhu J, Xiao D (2016). Antiferromagnetic spin wave field-effect transistor. Sci. Rep..

[10] Balinskiy M, Chiang H, Khitun A (2018). Realization of spin wave switch for data processing. AIP Adv..

[11] Schneider T, Serga AA, Leven B, Hillebrands B (2008). Realization of spin-wave logic gates. Appl. Phys. Lett..

[12] Jamali M, Kwon JH, Seo S, Lee K, Yang H (2013). Spin wave nonreciprocity for logic device applications. Sci. Rep..

[13] Shibata K, Kasahara K, Nakayama K, Kruglyak VV, Aziz MM, Manago T (2018). Dependence of non-reciprocity in spin wave excitation on antenna configuration. J. Appl. Phys..

[14] Kwon JH, Yoon J, Deorani P, Lee JM, Sinha J, Lee K, Hayashi M, Yang H (2016). Giant nonreciprocal emission of spin waves in Ta/Py bilayers. Sci. Adv..

[15] Demidov VE, Kostylev MP, Rott K, Krzysteczko P, Reiss G, Demokritov SO (2009). Excitation of microwaveguide modes by a stripe antenna. Appl. Phys. Lett..

[16] Nakayama M, Yamanoi K, Kasai S, Mitani S, Manago T (2015). Thickness dependence of spin wave nonreciprocity in permalloy film. Jpn. J. Appl. Phys..

[17] Sadovnikov AV, Beginin EN, Sheshukova SE, Sharaevskii YuP, Stognij AI, Novitski NN, Sakharov VK, Khivintsev YuV, Nikitov SA (2019). Route toward semiconductor magnonics: Light-induced spin-wave nonreciprocity in a YIG/GaAs structure. Phys. Rev. B..

[18] Chen J, Yu T, Liu C, Liu T, Madami M, Shen K, Zhang J, Tu S, Alam MS, Xia K, Wu M, Gubbiotti G, Blanter YM, Bauer GEW, Yu H (2019). Excitation of unidirectional exchange spin waves by a nanoscale magnetic grating. Phys. Rev. B..

[19] Deorani P, Kwon JH, Yang H (2014). Nonreciprocity engineering in magnetostatic spin waves. Curr. Appl. Phys..

[20] Gallardo RA, Schneider T, Chaurasiya AK, Oelschlägel A, Arekapudi SSPK, Roldán-Molina A, Hübner R, Lenz K, Barman A, Fassbender J, Lindner J, Hellwig O, Landeros P (2019). Reconfigurable spin-wave nonreciprocity induced by dipolar interaction in a coupled ferromagnetic bilayer. Phys. Rev. Appl..

[21] Albisetti E, Tacchi S, Silvani R, Scaramuzzi G, Finizio S, Wintz S, Rinaldi C, Cantoni M, Raabe J, Carlotti G, Bertacco R, Riedo E, Petti D (2020). Optically inspired nanomagnonics with nonreciprocal spin waves in synthetic antiferromagnets. Adv. Mater..

[22] Demokritov SO, Demidov VE (2008). Micro-Brillouin light scattering spectroscopy of magnetic nanostructures. IEEE Trans. Magn..

[23] Stamps R, Camley R (2014). Solid State Physics.

[24] Kalinikos BA, Slavin AN (1986). Theory of dipole-exchange spin wave spectrum for ferromagnetic films with mixed exchange boundary conditions. J. Phys. C Solid State Phys..

